# Attitudes, perceptions and knowledge among men who have sex with men towards the blood donation deferral policy in Israel

**DOI:** 10.1371/journal.pone.0170364

**Published:** 2017-02-02

**Authors:** Itzchak Levy, Liraz Olmer, Yuval Livnat, Adir Yanko, Eilat Shinar

**Affiliations:** 1 HIV Unit, Infectious disease unit, Sheba medical center, Ramat Gan, Israel; 2 Gertner institute for epidemiology and health policy research, Sheba Medical Center, Ramat Gan, Israel; 3 Israeli AIDS Task Force, Tel Aviv, Israel; 4 Magen David Adom national blood services, Ramat Gan, Israel; McGill University AIDS Centre, CANADA

## Abstract

**Background:**

Men who have sex with men (MSM) are permanently deferred from donating blood in Israel. Pressure to change this policy exists, despite data showing higher prevalence and incidence of HIV in MSM. A survey was conducted to evaluate current knowledge, attitudes, perceptions and compliance if deferral was changed.

**Study design and methods:**

Anonymous survey was published in a gay-oriented website, collecting demographic information, history of blood donation, attitudes, knowledge and compliance with permanent versus temporary deferral. Responses were analyzed given 1 point for every "yes" response (0–7 points). Student’s t-test was applied to compare differences between continuous variables. Correlations were described with the Pearson correlation coefficient.

**Results:**

Responses from 492 MSM were analyzed. Average age was 31±9 years. 76% donated blood at least once, mostly for social solidarity (score of 3.2 on 1–5 scale). Tests seeking or protest scores were 1.7 and 1.6, respectively. 66% were unaware of the higher risk of HIV transmission by MSM, or the potential to infect 3 recipients. Knowledge regarding HIV transmission by blood positively correlated with knowledge regarding other routes of HIV transmission (r = 0.11; p = 0.03), age (r = 0.10; p = 0.04), and higher rate of non-compliance with the current deferral policy (OR = 1.9; p = 0.02). Activism for LGBT rights was associated with lower risk for non-adherence (OR = 0.5; p = 0.03). If temporary deferral is introduced 66% will comply with the new policy, but 23% will continue to donate as long as MSM deferral policy is in place.

**Conclusion:**

A high proportion of MSM do not comply with the current lifetime deferral. This may partially change if temporary deferral is introduced.

## Introduction

Since the very early days of the HIV epidemic the Israeli Ministry of Health (MOH) followed international regulatory authorities introducing permanent deferral of men who had sex with men (MSM) since 1977 from donating blood [1–3].

According to the national Standard Operation Procedures (SOP) volunteers who wish to donate blood must complete a written Donor Health Questionnaire (DHQ), specifying medical conditions and situations/behavior that may lead to self-deferral. This is followed by a discreet interview performed by a professional phlebotomist.

Unlike many countries, the Israeli DHQ does not contain direct questions regarding the potential donors' sexual orientation or behaviors as used in other countries. Instead, in the DHQ (S1 Appendix, questions 2.5–2.13; 2.15) specific situations of high risk behavior are mentioned, and donors are informed that if any of those are is applicable, the donated unit cannot be used for transfusion. Donors who find the information relevant should either refer from donating or defer themselves by marking question 2.14 as "not for transfusion", without specifying the reason for such deferral [4].

In Israel, the annual incidence of newly diagnosed HIV patients ranges between 58.5–61 cases per million population [5]. At least 34% of the 8,000 diagnosed HIV patients living in Israel are MSM. According to the ministry of health there are at least 2000 undiagnosed patients [5].

Based on the Israeli National Blood Services (Magen David Adom -MDA) database, 72% of the 1.2 million volunteer blood donors registered since 1987 were males. Of them, only 879 (0.07%), self—deferred due to high risk behavior. Of those who did not defer themselves, most of those of whom we have complete data (34/37) of male donors diagnosed as HIV positive following tests of the donated unit, declared being MSM, hence they did not comply with the current deferral policy (Shinar and Levy, personal communication).

In the last years pressure from the LGBT communities and the Israeli AIDS Task Force call for changes of the current deferral policy. This has been based on replacement of the permanent by temporary deferral policy of MSM in Europe, Canada and Australia [6–9] and on the recent FDA guidelines, published in December 2015, where the donor deferral policy for MSM was changed to a 1-year deferral from last sexual contact [10].

In addition, the Israeli Ministry of Health (MOH) appointed an ad-hoc independent committee to determine if changes in the policy can be adopted, hoping that introduction of a temporary deferral, according to a defined abstinence period, may help to deal with the MSM perception of discrimination and rejection, and lead to better compliance with the national policy, without significantly compromising blood safety.

Having in mind that shortening the deferral time increases the dependence on the compliance of MSM with the new policy and, previous observations documenting non-compliance with current deferral criteria among MSM whose behavioral risks should have precluded them from blood donation [11–14], a survey was conducted among MSM, using an on-line questionnaire, to understand attitudes, knowledge, behavior and compliance regarding blood donation and transfusion recipients' safety.

## Methods

A 21-questions anonymous survey was published in a gay-oriented website (http://dating.atraf.co.il) from 19 May 2014 to 19 June 2014, asking respondents for data regarding demographic information, history of blood donation in Israel, knowledge, and attitudes towards permanent versus temporary deferral of MSM. Inclusion criteria were being a male older than 18 years of age, MSM orientation and residing in Israel. HIV-positive participants were excluded from analysis. Participants were asked to suggest how the current deferral policy should be modified and if their compliance will be different upon changing the policy. Participants were requested to respond only once as a means of reducing sampling bias. No financial incentive was offered.

The survey questionnaire (S2 Appendix) included three categories: (i) demographic information, including age, status and level of education; (ii) history of blood donation in Israel; (iii) knowledge and attitudes towards permanent *vs*. temporary deferral of MSM.

The last part included 6 items regarding HIV risk and blood donation and 7 items regarding knowledge concerning HIV transmission among MSM.

After replying to the questions regarding HIV risk and blood donation, participants were asked to answer again the questions regarding their attitude to and compliance with the current deferral policy. All Responses were analyzed given 1 point per "yes" response (the range of possible scores was 0–7 points).

Analyses were performed using SAS software. Means ± SD were calculated for continuous variables, and absolute and relative frequencies were measured for discrete variables. Student’s t-test was applied to compare differences between continuous variables. Correlations were described with the Pearson correlation coefficient. Univariate analyses were performed to test for associations between non-compliance (dependent variable) and various covariates. Covariates found to be associated with the dependent variable were candidates in the stepwise multivariate logistic regression analysis, performed to identify predictors of non-compliance. Odds ratios (OR) and 95% confidence intervals (CI) were calculated in the final model. All tests of significance were two tailed. A value of P<0.05 was considered statistically significant.

### Ethical approval

Since this as an anonymously online survey an individual verbal or written consent were not obtained. Responders were informed in the beginning of the survey that they may withdraw from the questionnaire at any time.

Ethical approval including this consent procedure (IRB 0413-13-SMC) was obtained from the Institutional Review Board (IRB) of Sheba Medical Center prior to implementation of the study.

## Results

### Characteristics of the sample

**Fig 1:**
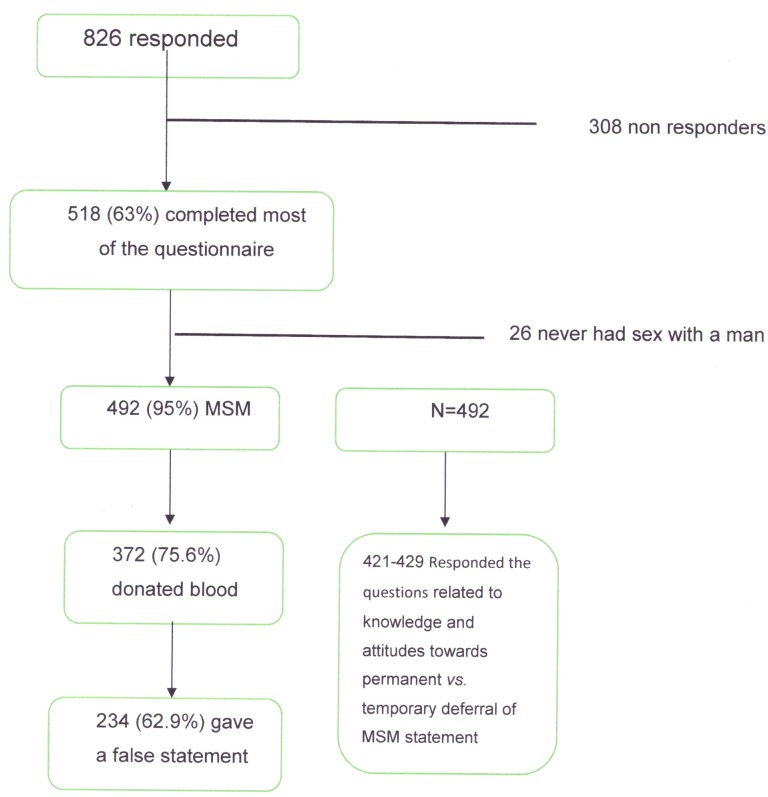
Participants flow diagram.

Sixty three per cent (518/ 826) of the persons responded to the online survey questionnaire and completed most of the 21 questions (Fig 1). Of them 507 were men, of whom only 492 were included in the final analysis of the survey, as they defined themselves as MSM.

Table 1 depicts the general characteristics of the participating population. For those reporting their age, 299 (70%) were between 18–33 years old, average age of 31±9 years. About 67% were single, and 83% had high-school or higher education.

**Table 1:** Demographic characteristics.

Characteristics	N	%
**Age**		
18–25	141	28.7
26–33	158	32.1
34+	126	25.6
Did not answer	67	13.6
**Marital status**		
Single	330	67.1
In relationship	57	11.6
Married to a man	23	4.7
Married to a woman	11	2.2
Divorced/widowed	6	1.2
Did not answer	65	
**Education**		
0–11 years	18	3.7
12 years (high school)	150	30.5
BA	178	36.2
MA and higher	81	16.5
Did not answer	65	13.2

### History of and reasons for blood donations

As mentioned above, potential blood donors in Israel are not requested to identify themselves as MSM to the interviewer, but rather mark the "non-for transfusion" box in the DHQ. However, for the sake of clarity and understanding by the survey respondents, non-compliance with this requirement was defined as "making a false statement" regarding their sexual orientation. This terminology is also used in the analysis of the results.

About 76% (372/492) of the survey MSM participants gave a history of previous blood donation in Israel, of whom 234 (63%) admitted making a false statement regarding their sexual orientation in the pre- donation DHQ and interview.

**Fig 2:**
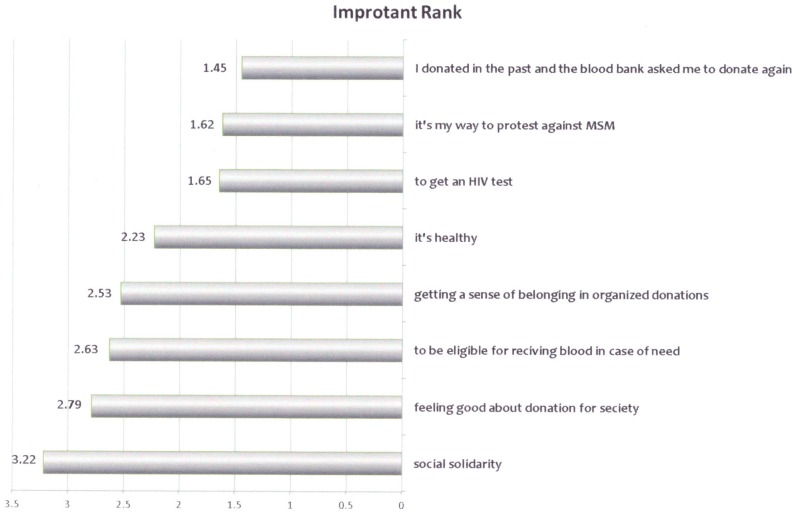
Reasons for blood donation. In this figure the reasons for blood donation according to their importance for the individual donor is shown.

When asked to score the reasons for donating blood by their importance (on a 1–5 scale, Fig 2), the leading cause was social solidarity (397/480 (83%) rated it as very important or important), followed by feeling of a highly meaningful deed / minimal time invested (328/478 (69%) rated it as very important or important). The reasons that were rated with the lowest rank were the desire to obtain HIV test results and as a protest against the attitude of the general population toward the LGBT community.

### Knowledge regarding risk of HIV transmission

The 6 items questionnaire regarding knowledge on the risk of HIV transmission via blood donation was answered by 420 men, and the average score ± SD (range) was 2.8±1.6 (0–6).

The 7 items part, regarding knowledge concerning other routes of HIV transmission was answered by 416 men and the average score ± SD (range)) was 5.2± 0.9 (2–7).

Among the 372 MSM who donated blood in the past the average score among those who did not comply with the current MOH policy was higher compared to those who did comply and deferred themselves (5.3±0.9 vs. 5.0±0.9, p = 0.04).

A positive correlation was found between knowledge regarding HIV risk in blood donation and knowledge concerning other routes of HIV transmission (r = 0.11; p = 0.03) as well as between knowledge regarding HIV risk in blood donation and age (r = 0.10; p = 0.04). There was no correlation with education (r = 0.03; p = 0.6).

The odds ratios (OR) were calculated to evaluate factors which may be associated with non-compliance with the current MOH policy. In univariate (Table 2) and multivariate (Table 3) analyses, higher knowledge about HIV was associated with a higher risk of non-compliance, whereas activism for LGBT rights was associated with higher compliance and a lower risk to give a false statement.

**Table 2:** Parameters associated with non-compliance—univariate by logistic regression.

Variable	Effect	Odds Ratio	Lower 95% CL	Upper 95% CL	P-value
Age	age 18–25 vs 34+	1.61	0.91	2.83	0.1026
Age	age 26–33 vs 34+	1.62	0.95	2.75	0.0759
NO single		1.13	0.66	1.92	0.6635
Academic degree		1.10	0.69	1.75	0.6854
LGBT Activism		0.54	0.29	0.97	0.0405
Not Agree with current policy		1.18	0.55	2.51	0.6731
HIV knowledge		1.95	1.15	3.30	0.0125
Donation knowledge		1.09	0.67	1.78	0.7153

**Table 3:** Parameters associated with non-compliance—multivariate model by logistic regression.

Variable	Odds Ratio	Lower 95% CL	Upper 95% CL	P-value
HIV knowledge	1.88	1.10	3.21	0.0217
LGBT Activism	0.47	0.23	0.94	0.0329

### Attitudes among MSM towards deferral from blood donation after answering the questionnaire on knowledge regarding the risk of HIV transmission in blood donation

About 87% (426/492) responded to the survey request, to answer once again the questions regarding their attitude to and compliance with the current deferral policy after reading and replying to the questions regarding the risk for HIV transmission in blood donation, as mentioned in the Method section. Not all participants responded to all the questions.

About 40% (171/426) said the information provided caused them to think differently regarding blood donation from MSM. When asked what would they change in the current phrasing of the DHQ, 61% (153/249) thought deferral should include any individual involved in un-safe sex (both MSM and heterosexuals), 20% said MSM should not defer themselves at all, 11% said that the deferral period should be shortened and adopted to the test "window period", while 3% responded that this should be done with no specific relation to the "window period".

After reading the "knowledge on HIV transmission" questionnaire, 135 of the 234 (57.7%) who did not comply with the current deferral policy and donated blood in the past declared they might change their behavior if current deferral policy will be changed. 66% indicated that they may comply with a new policy and 7.4% would defer themselves from donating blood as long as they practice sex with men. However, 27% would continue to donate blood and without self-deferral due to their sexual orientation as long as the deferral policy includes MSM.

## Discussion

As part of the decision-making process to change the current national policy of permanent deferral from blood donation of men who were engaged in sex with other men (MSM) since 1977, a close collaboration was created among all the stakeholders involved in HIV prevention in the country, including the performance of an on-line survey, with the intention to learn more on attitudes, perceptions, knowledge and possible compliance among MSM. The survey revealed that 76% of MSM who responded to the questionnaire donated blood at least once, and did not consider to refrain or to use the self-deferral option.

This high proportion of participating individuals who report donation seems unusually high compared to other studies but the higher percentage of males among the blood donor population in Israel (72%), when compared to about 50% in the USA or Europe should be taken into account [15, 16].

The reasons for such a relatively high percentage of non-compliance of MSM are of concern, when compared to those reported by others [17]. It should be taken into account that our survey is somewhat different from other studies that examined this question inasmuch as most studies have been focused upon individuals who have donated blood and only subsequently acknowledged MSM behavior where as our study was directed towards MSM only asking them about past and future behavior regarding blood donation.

One possible explanation could be due to the way blood drives are organized and donations are perceived in Israel: Over 95% of the donated units are collected in organized mobile, blood drives in high schools, work places or in the military. In most places people belonging to the group go together to the blood drive, and are usually encouraged and influenced by the attitude of significant person (superior or commander) to donate [18]. It may therefore be difficult to resist donation and be an "outsider", or even be linked to “coming out” and the fear of stigma or discrimination may prevent disclosure. The fact that the most important reasons for donating blood chosen by the survey responders were "feeling part of the general community" (social solidarity) and "feeling part of the private community" (such as work, school or army), while "test- seeking" and blood donation as a way to protest against the current policy were both ranked low may support this assumption.

In addition, Israel is captured by many of its citizens as an isolated country, especially in time of emergency, so it is conceivable that people, including the survey participants, perceive blood donation as an act of patriotism. In addition, a selection bias may be considered, due to a possible over- representation of individuals who wish to, and actually donated blood in the survey.

We therefore believe that further investigation should be conducted to identify the reasons for such a significant non-compliant behavior, comparing local aspects (i.e. social, political, etc.) in the Gay community in Israel to those in other countries, including the observations that higher knowledge about HIV was associated with a higher risk of non-compliance, whereas being an LGBT activist decreased the odds for non-compliance. Most participants shared good knowledge regarding HIV transmission among MSM, but not regarding transmission through blood transfusion, with 64% unaware of the higher risk of HIV transmission in blood donated by MSM in Israel, when compared to the general donor population.

Our finding that those with higher knowledge regarding HIV transmission are more likely to improperly donate blood is not entirely understood and may be even counterintuitive. One explanation may be that people with higher knowledge may be with higher confidence regarding the safety of their sexual behavior. One study showed that 43.7% of MSM that donated blood inappropriately in Hong Kong did so because they felt confident regarding the safety of their blood at the time of donation [17]. It is interesting to note that in a recently published study it was actually found that the overall prevalence of HIV infection among noncompliant males that inappropriately donated blood in the USA was much lower than the overall rates of HIV infection among MSM [19]. The authors suggest that noncompliant donors may understand their risk status and that those that do comply defer themselves from donation which may implicate some degree of self-selection.

Although the theoretical risk of HIV transmission in blood transfusion is considered extremely low and estimated to be no more than 1 to 1.5–2 million donations due to the current good testing methods [20–23], improving donors' compliance is of outmost importance, especially if a change in the policy is considered. An important observation in the survey was that two thirds of the MSM who did not comply with the current deferral policy and donated blood said that if a temporal deferral based on an abstinence period will be introduced they will comply with it, or will even defer from donating blood as long as they are sexually active.

The association between LGBT activism and higher compliance with blood donation deferral policies was not studies before and indeed we don't have a valid explanation for this. One possible theory may be that individuals that are "in the closet" are less active in the LGBT community and use more often the blood donation for HIV tests or are less knowledgeable regarding the risk of HIV transmission through blood donation. Off course it may be that some of them do not connect themselves emotionally and mentally as belonging to the MSM group and therefore do not refer to themselves as belonging to a risk group and thus they do not defer themselves from donation. In fact, in our clinic from the 37 HIV + patients that were discovered through blood donation 34 were MSM, 20 of them (59%) did not define themselves as homosexuals and most (16/20) were married to women and never revealed their MSM relationship to their spouses. When asked later they say they did not considered themselves as belonging to a risk group. Individuals active in the LGBT society may be more knowledgeable and more aware to the risk they may impose by blood donation and thus comply more with self-deferral policies.

While this study depicts the Attitudes, perceptions and knowledge among men who have sex with men towards the blood donation deferral policy in Israel for the first time, it is subject to several limitations. First, the MSM that were recruited through the websites may not be representative of the larger community of MSM. Conducting an online study may have different drawbacks, especially regarding sampling [24]. The information collected through an online survey may be misleading since responders may not answer accurately and the data accepted cannot be verified and thus may be questionable. Second, self-selection bias is a major limitation of online survey research. There are undoubtedly some individuals who were more likely than others to enter "gay websites" and complete our survey. As one of the main purposes of these sites are meeting people for sexual encounters this may cause a bias towards more MSM that are single than in partnership to enter and respond. Third, the study is subject to reporting or to recall bias. Fourth, the results of this study are limited by the small sample size. Fifth, it may be possible that participants were biased towards more socially acceptable answers to at least some of the questions, especially since around the time the questionnaire was presented discussion concerning self-deferral among MSM were begun in the media. Sixth, the questionnaire was available in Hebrew only, thus missing individuals who were not able to read the questions individually. In a country like Israel where about 20% of its inhabitant are immigrants this may be a problem. Lastly, due to the cross-sectional nature of our study one must consider the issue of temporality. It should be noted that If blood donation preceded first MSM experience than the donor is not making a false statement on the DHQ, but still is considered to give a false statement in our survey. Unfortunately we don't have the data for all the different possible combinations of possible answers and thus cannot provide actual self-deferral after having MSM contact. Nevertheless, due to the nature of the web site that the survey was published we can safely state that most of the participants had already had MSM contact while answering the survey.

These limitations inhibited our ability to make generalizations about study findings to the general MSM population in Israel.

In summary, a very high proportion of MSM in this study admitted donating blood while showing noncompliance with the current permanent deferral policy. The act of blood donation is done regardless of relatively adequate knowledge about HIV transmission. However, when additional relevant information was delivered to the survey participants, about two thirds considered changing their attitude, and comply better, if a reasonable deferral-policy be introduced.

We recommend that implementation of a temporary deferral policy in Israel should be accompanied by education of potential donors regarding accurate self-evaluation of their risks and appropriate self-deferral. Major efforts should be dedicated to studying and changing the attitude, knowledge and compliance among MSM. Involvement of the MSM community NGOs in these processes is of utter importance.

In view of the high number of the survey participants, who declared they will not comply with any deferral policy, The establishment of a national Hemovigilance system is of outmost importance, as it will allow monitoring of the national blood program, and evaluation of the impact of the introduced changes on maintaining blood safety and quality In Israel.

## Supporting information

S1 Appendix Blood donor health questionnaire. (DOCX)

S2 Appendix The survey questionnaire. (DOCX)
