# Supplementary figures and images for: Attitudes, perceptions and knowledge among men who have sex with men towards the blood donation deferral policy in Israel
Source: PLoS One. 2017 Feb 2;12(2):e0170364. doi: 10.1371/journal.pone.0170364 (PMC5289429; doi:10.1371/journal.pone.0170364)

Appendix 1: Blood Donor Health Questionnaire


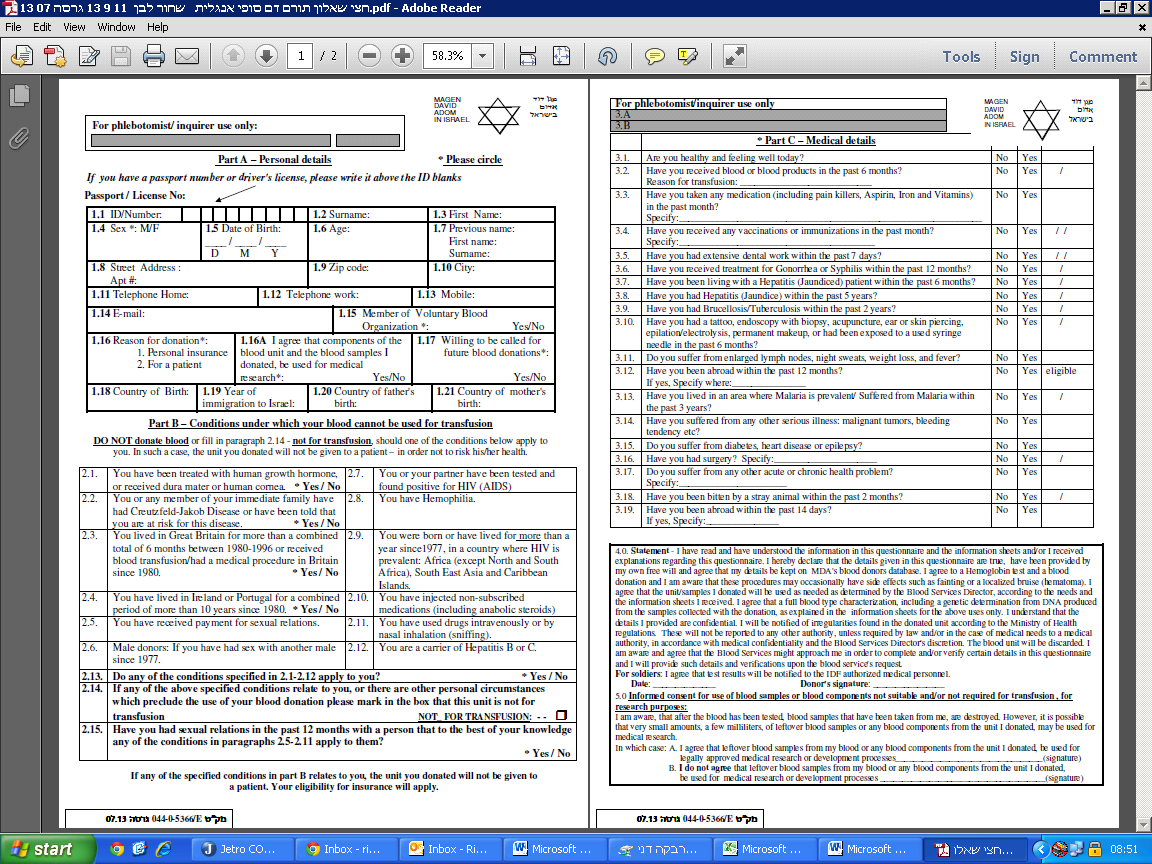

Supplement: S1 Appendix — (DOCX) [file pone.0170364.s001.docx]

Appendix 2: Survey


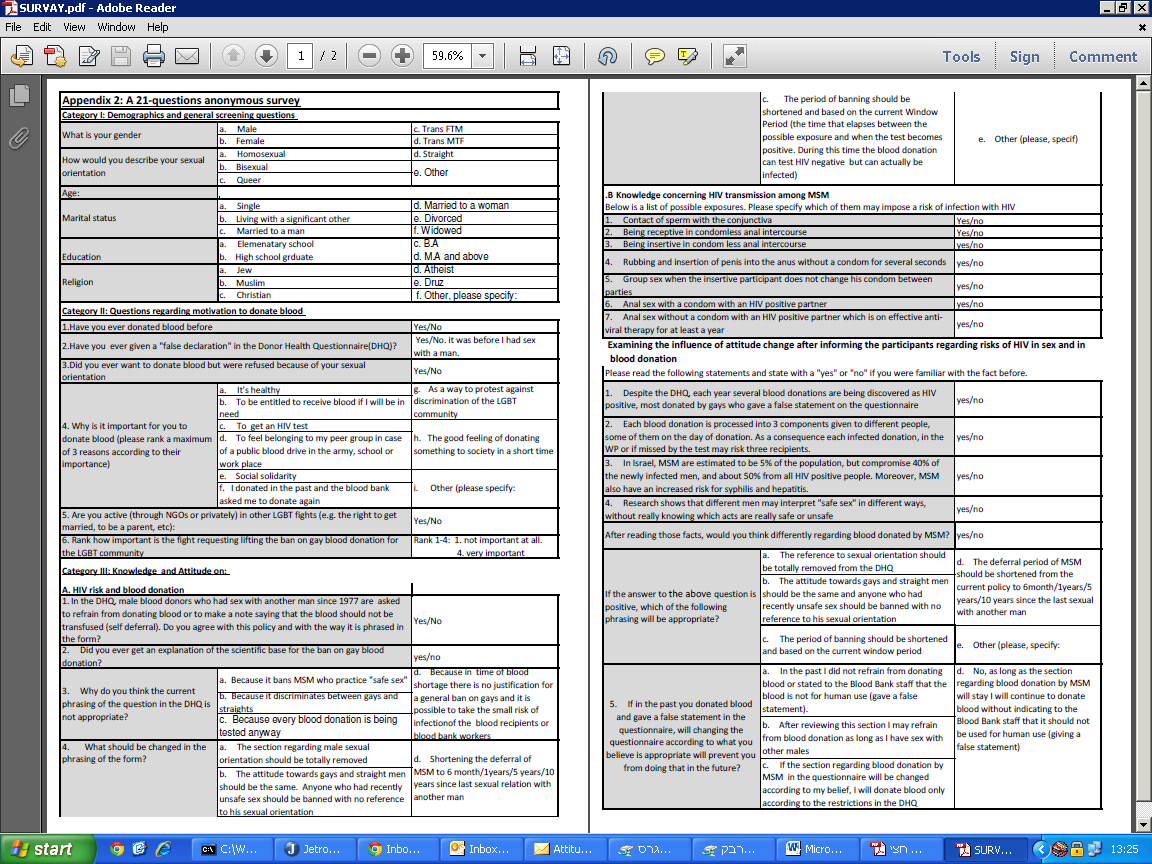


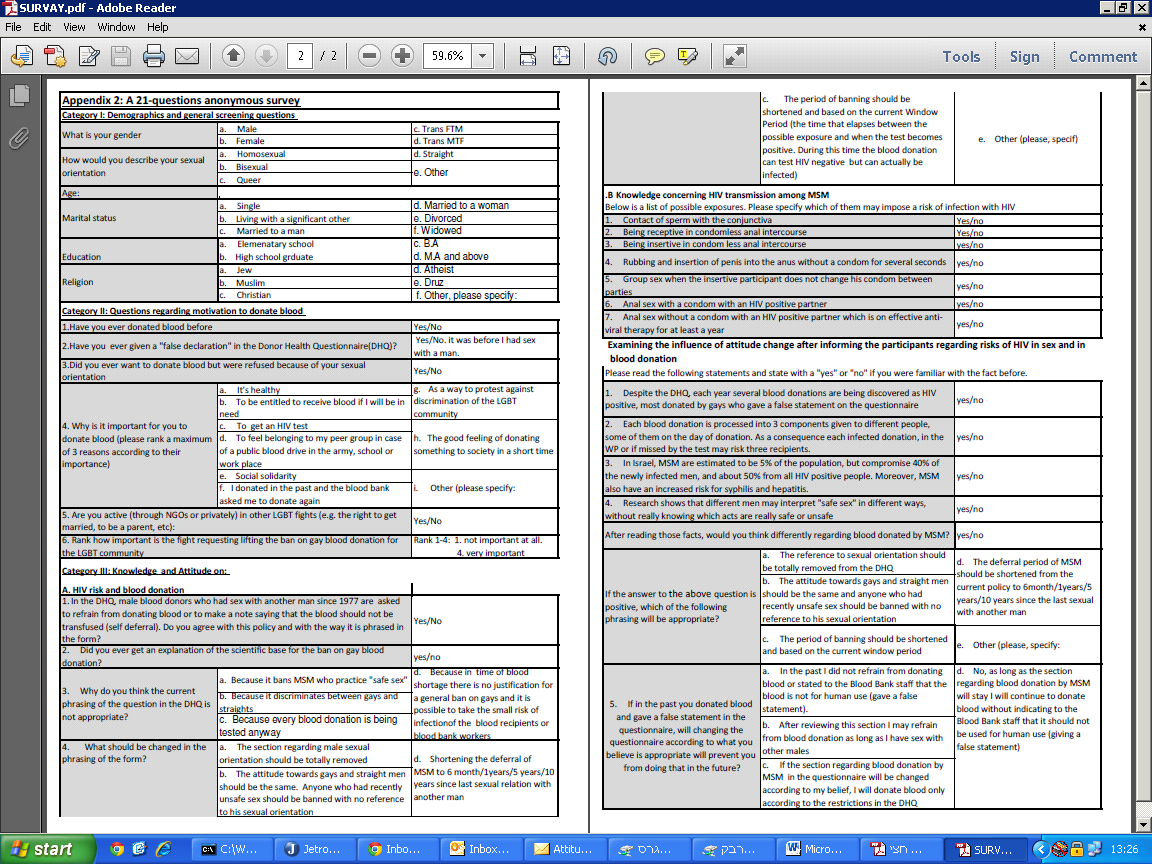

Supplement: S2 Appendix — (DOCX) [file pone.0170364.s002.docx]
